# Authors’ Reply: Is the Pinball Machine a Blind Spot in Serious Games Research?

**DOI:** 10.2196/73034

**Published:** 2025-04-02

**Authors:** Luis Carlos Rodríguez Timaná, Javier Ferney Castillo García, Teodiano Bastos Filho, Alvaro Alexander Ocampo González, Nazly Rocio Hincapié Monsalve, Nicolas Jacobo Valencia Jimenez

**Affiliations:** 1 Faculty of Engineering, Universidad Santiago de Cali, Calle 5 # 62-00 - Pampalinda, Santiago de Cali, 763022, Colombia, 57 3234664283; 2 Faculty of Engineering, Universidad Autónoma de Occidente, Cali, Colombia; 3 Faculty of Engineering, Universidade Federal do Espírito Santo, Espiritu Santo, Brazil; 4 Faculty of Health, Universidad Santiago de Cali, Santiago de Cali, Colombia

**Keywords:** serious games, research, interventions, arcade technology, digital game paradigm, pinball gaming, arcade gaming, executive functions, neurodiversity, cognitive training, therapeutic interventions

We appreciate the insightful comments and reflections from the author of the letter [1] regarding our study on the impact of serious games on executive functions and their application in neurodiverse populations [2]. The suggestion to consider pinball machines as a tool within the serious games paradigm presents an interesting avenue for further exploration.

At the time of our study, our focus was primarily on conventional and emerging digital technologies, such as virtual reality, mobile devices, and sensor-based interactions. However, we acknowledge that pinball, with its unique combination of physical and digital interactions, may offer valuable cognitive and therapeutic benefits, particularly in the context of executive function training. The references provided in the letter [1] highlight historical and recent research supporting its potential applications in various populations, reinforcing the idea that this arcade technology could play a role in future serious game developments.

Given the evidence presented on pinball’s ability to engage attention, impulse control, cognitive flexibility, and problem-solving skills, we recognize its potential as a tool to enhance executive function training. Future work in this area could explore the adaptation of pinball mechanics within digital serious games or investigate its direct application as a therapeutic tool in controlled settings.

Additionally, we acknowledge that the development of assistive technologies for neurodiverse populations often encounters blind spots, where certain tools or approaches are overlooked. Our intention with the published paper is to provide a road map for researchers, highlighting that there remains substantial work to be done in this area. By identifying these gaps, we aim to offer a starting point for ongoing and future investigations.

Several studies have underscored the challenges and opportunities in designing technologies for neurodiverse users. For instance, Frauenberger et al [3] discuss the importance of involving neurodiverse children in the technology design process to ensure that their unique needs are met. Similarly, Benton and Johnson [4] highlight lessons from neurodiverse communities, emphasizing the necessity of tailored technological interventions. These perspectives reinforce the need for comprehensive research and development efforts to address the diverse requirements of neurodiverse populations.

We thank the author of the letter [1] for broadening the discussion on serious game technologies. Their insights open the door to new interdisciplinary research possibilities that could further enrich this field.
